# Implementing harm reduction kits in an office-based addiction treatment program

**DOI:** 10.1186/s12954-023-00897-5

**Published:** 2023-11-02

**Authors:** Margaret Shang, Brent Thiel, Jane M. Liebschutz, Kevin L. Kraemer, Ariana Freund, Raagini Jawa

**Affiliations:** 1 Division of General Internal Medicine, Department of Medicine, University of Pittsburgh School of Medicine, Pittsburgh, PA USA; 2 Center for Research on Healthcare, University of Pittsburgh School of Medicine, 3609 Forbes Ave, Pittsburgh, PA 15213 USA

**Keywords:** Harm reduction, Safer use equipment, Office-based addiction treatment, Substance use disorder, Addiction

## Abstract

**Background:**

The rising rates of drug use-related complications call for a paradigm shift in the care for people who use drugs. While addiction treatment and harm reduction have historically been siloed in the US, co-location of these services in office-based addiction treatment (OBAT) settings offers a more realistic and patient-centered approach. We describe a quality improvement program on integrating harm reduction kits into an urban OBAT clinic.

**Methods:**

After engaging appropriate stakeholders and delivering clinician and staff trainings on safer use best practices, we developed a clinical workflow for universal offering and distribution of pre-packaged kits coupled with patient-facing educational handouts. We assessed: (1) kit uptake with kit number and types distributed; and (2) implementation outcomes of feasibility, acceptability, appropriateness, and patient perceptions.

**Results:**

One-month post-implementation, 28% (40/141) of completed in-person visits had at least one kit request, and a total of 121 kits were distributed. Staff and clinicians found the program to be highly feasible, acceptable, and appropriate, and patient perceptions were positive.

**Conclusions:**

Incorporating kits in OBAT settings is an important step toward increasing patient access and utilization of life-saving services. Our program uncovered a significant unmet need among our patients, suggesting that kit integration within addiction treatment can improve the standard of care for people who use drugs.

**Supplementary Information:**

The online version contains supplementary material available at 10.1186/s12954-023-00897-5.

## Background

The rate of overdose deaths in the US has reached an unprecedented high, fueled initially by the increased prescription of opioids in the early 1990s [1, 2]. Over time, the US illicit drug supply has transitioned to progressively more potent and toxic substances including non-pharmaceutical fentanyl analogs and other adulterants like xylazine [2, 3]. This shift combined with high rates of polysubstance use has also contributed to rising rates of other drug-related harms including infectious complications like hepatitis C, infective endocarditis, and skin and soft tissue infections [4–6]. The increasing public health impact of illicit drug use calls for a paradigm shift in our approach to caring for people who use drugs (PWUD) [7].

Harm reduction services, which are usually offered at community-based syringe service programs (SSPs), include providing sterile drug use equipment, fentanyl test strips, overdose education and prevention, wound care, laboratory testing, and linkage to treatment. Numerous studies have demonstrated the significant positive impact of SSPs on patient and population-level outcomes [8, 9]. SSPs, however, are not universally accessible in the US as a result of complex regional regulations [10] and so expanding these services to healthcare settings may increase access to harm reduction, be a cost-effective strategy [11], and improve clinical outcomes among PWUD [12].

Primary care treatment settings including office-based addiction treatment (OBAT) programs [13] serve as an opportune venue for the integration of addiction treatment and harm reduction. While these services have historically been siloed in the US and viewed as having distinct and incompatible goals [14, 15], co-location offers a compassionate, pragmatic, and patient-centered approach to care for PWUD. It also acknowledges the reality that while medications for opioid use disorder (MOUD) such as buprenorphine and methadone are evidence-based treatments that reduce opioid-related risks [16–19], patients may continue to use illicit substances [20, 21].

Exploring strategies to integrate harm reduction in healthcare settings aligns well with US national priorities as evidenced by the 2021 Biden–Harris National Drug Control Strategy and 2023 Health Resources and Services Administration’s guidance allowing Federally Qualified Health Centers to offer SSP services [7, 22], as well as international efforts advocating for implementing harm reduction-based care models such as the incorporation of an overdose prevention site in the hospital setting [23–25]. However, while strategies to incorporate harm reduction into outpatient primary care settings in the US have been recommended in prior work [26, 27], practical implementation methods have not been well described. To fill this critical gap, we describe the process of implementing a quality improvement (QI) program focused on integrating harm reduction kits (referred to as kits hereafter) into an urban OBAT clinic.

## Methods

### Setting and participants

The Internal Medicine-Recovery Engagement Program (IM-REP) is an OBAT program at the University of Pittsburgh Medical Center (UPMC) which is designated as one of Pennsylvania’s Centers of Excellence for Opioid Use Disorder. The clinic has a multidisciplinary team of addiction medicine-trained generalist physicians, certified recovery specialists, nurses, mental health therapists, a medical receptionist, and an administrative coordinator. In 2022, the clinic served 375 unique patients (81.3% non-Hispanic white, 16.5% non-Hispanic Black) who have a primary substance use disorder (SUD) diagnosis and may have co-occurring behavioral and physical health conditions. Services provided at our clinic include evaluation and treatment of SUD including MOUD, primary care including hepatitis C treatment, behavioral health and psychiatric services, social services support, and peer recovery support. We have a hybrid clinic structure with approximately half in-person and half telemedicine visits.

**Fig. 1:**
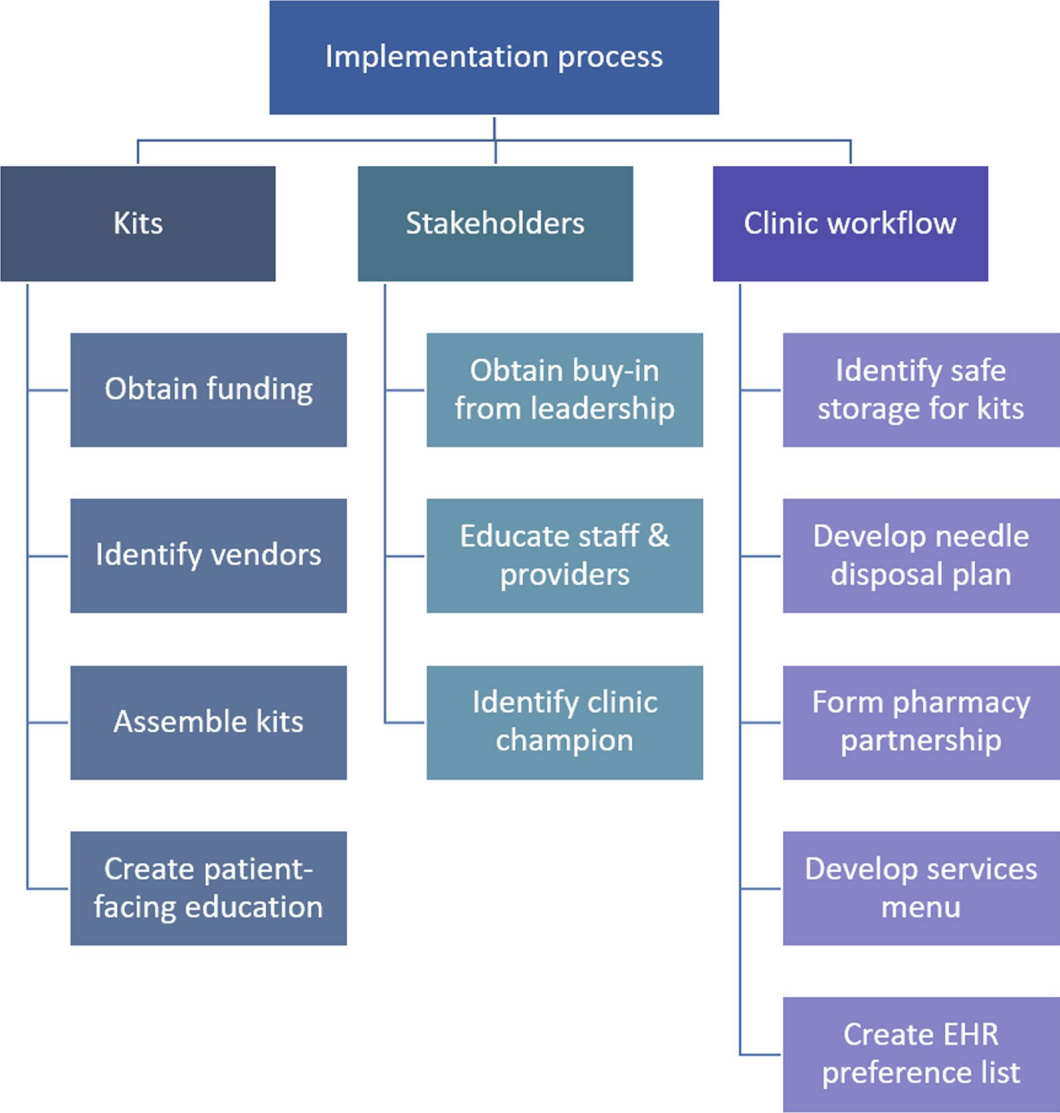
Suggested steps for harm reduction kit pre-implementation in an office-based addiction treatment clinic. Abbreviations: EHR, electronic health record

Our QI program was approved by the UPMC Quality Improvement Review Committee and deemed exempt from further review by the University of Pittsburgh Institutional Review Board as it was not considered human participant research. Figure 1 provides an overview of the program’s planning and implementation process.

### Pre-implementation

#### Obtaining clinic stakeholder support

Our team initially identified departmental and clinic administrative leadership stakeholders and sought their buy-in for the program. Next we conducted stakeholder meetings to describe the importance of harm reduction in OBAT settings in averting drug-related harms and discuss program goals to increase patient engagement in addiction treatment. Lastly, we obtained departmental pilot funding to cover the initial costs required for kit supplies.

#### Staff training and assessment

Given that our multidisciplinary clinic staff have variable backgrounds and experiences related to addiction treatment and harm reduction, we delivered an introductory one-hour training during a weekly staff meeting in December 2022. This training reviewed harm reduction principles, drug overdose and naloxone education, and safer use best practices. Staff completed a ten-question de-identified Qualtrics training survey immediately before and after the training to assess their knowledge and comfort related to harm reduction practices. Knowledge questions were multiple choice or true/false and assessed staff knowledge about harm reduction such as “what is the most common bloodborne viral infection among individuals who use drugs?” Staff were asked about comfort related to naloxone administration, kit distribution, and correct kit selection, all of which were measured using a 5-point Likert scale (1 = very uncomfortable, 5 = very comfortable). Pre- and post-training comfort items were compared using Wilcoxon signed-rank test.

Ten staff completed the training and pre/post-training survey. We observed an increase in staff knowledge across almost all domains as well as in average Likert score for all comfort measures (Additional file 1: Table S1). Based on these results as well as direct feedback from clinic staff, we provided two additional hour-long sessions. The first focused specifically on overdose response and prevention (i.e.: when and how to administer naloxone) and the second reviewed the evidence for safer use equipment and ways to coach patients on safer use drug practices in January 2023.

#### Kits and supplies

Informed by local SSP guidance and practices, we assembled safer injection, safer smoking, safer snorting, safer boofing, fentanyl test strips, and wound care kits. Table 1 provides details on the type, approximate cost, and contents for each kit with rationale for inclusion. We identified vendors for kit supplies and purchased enough inventory based on anticipated patient demand during our one-month pilot. Using the estimated cost per kit or item (see Table 1), the total cost for the number of kits distributed in one month was US$1,021. Needles were not included in our safer injection kits due to Pennsylvanian state laws criminalizing certain types of drug use equipment. As a workaround, if injection kits were requested, needles were prescribed to our on-site institutional pharmacy using electronic health record preference lists, which are personalized shortcuts for placing orders. For each kit, we developed complementary patient-facing educational pamphlets (see Additional file 1: Fig. S1), which included information about kit contents with their purpose and/or intended use, other harm reduction strategies related to kit type, and additional resources regarding safer drug use. Pre-implementation, clinic staff and clinicians helped to assemble kits as a group and familiarize themselves with kit contents and harm reduction strategies.

**Table 1:** Descriptions of the different types of harm reduction kits

Kit type	Cost/kit* (US$)	Kit contents	Rationale for inclusion [15, 30, 38–43]
Fentanyl test strips	$1.91	Cooker (bottle cap)	Reduces risk of infection by encouraging avoidance of sharing or reuse of cooker, particularly if the dissolved contents are injected afterwards
		Fentanyl test strip	Tests for fentanyl in drugs and may decrease risk of overdose by better informing PWUD before use, particularly for pressed pills, stimulants, and other non-opioid substances
		Sterile water (5 mL vial)	Used to dissolve and subsequently dilute drugs for testing, particularly if the dissolved contents are injected afterwards
Safer boofing^†^	$1.06	Cooker	Reduces risk of infection by encouraging avoidance of sharing or reuse of cooker
		Lubricant	Applied to tip of syringe before inserting into rectum. Reduces risk of damaging rectal mucosa
		Sterile saline (3 mL vial)	Used to dissolve drugs before per-rectal use
		Syringe (1 mL, Luer slip)	Used to draw up drugs for per-rectal use. Reduces risk of infection by encouraging avoidance of sharing or reuse
Safer injection	$6.08	Alcohol wipe	Used to clean skin before injecting and reduces risk of infection
		Ascorbic acid	Used to help dissolve certain drugs like crack cocaine for injection. Prevents use of vinegar or citrus fruits (lemon juice), which can increase damage to veins and risk for fungal infections (*Candida spp.*)
		Cooker	Reduces risk of infection by encouraging avoidance of sharing or reuse of cooker
		Cottons (dental)	Acts as a filter for undissolved solid particles such as the coating of crushed pills. Reduces risk of infection by encouraging avoidance of reuse of cotton
		Needles^‡^	Reduces risk of infections by preventing needle sharing or reuse
		Sharps container (Fitpack, fits up to 10 insulin syringes)	Reduce community presence of needles by allowing for safe disposal. Sleek understated design allows for discrete storage and use
		Sterile saline	Used to dissolve drugs and reduces risk of infection associated with use of boiled, bottled, or tap water
		Tourniquet	Allows for better visualization/palpation of veins to reduce multiple attempts at injecting. Decreases potential risk of vein damage and infection
		Twist tie	Used to fashion a handle for cooker. Reduces risk of burning fingers if applying heat source to cooker when preparing drugs for injection
Safer smoking (crack cocaine)^§^	$0.73	Alcohol wipe	Used to clean mouthpiece and pipe to reduce risk of infection
		Lip balm	Keeps lips moist to reduce cracks and blisters
		Mouthpiece	Helps to prevent cutting or burning lips on hot pipe
		Screen	Used as filter to keep crack rock in place. Prevents use of steel wool (Brillo®), in which hot pieces can break off and damage the mouth, throat, and lungs. Steel wool coating is also toxic
		Wooden push stick	Used to push screen into place. Wood is less likely than metal to damage the pipe
Safer smoking (methamphetamines)^§^	$0.59	Alcohol wipe	Used to clean mouthpiece and pipe to reduce risk of infections
		Lip balm	Keeps lips moist to reduce cracks and blisters
		Mouthpiece	Helps to prevent cutting or burning lips on hot pipe
Safer snorting	$0.47	Alcohol wipe	Used to keep surfaces clean to reduce risk of infections
		Blank card	Used as a clean surface to crush and snort drugs from
		Plastic razor	Used to crush drugs into a finer powder to reduce damage to nasal mucosa
		Plastic straws (different colors)	Reduces risk of infection by encouraging avoidance of sharing, reusing, or using dollar bills. Different colors allow individuals to keep track of their own equipment
		Scoop	Reduces risk of infection by preventing use of other contaminated objects like keys as scoops
Wound care	$6.49	Bandages	Used to cover wounds
		Burn cream	Used to apply to any burn wounds
		Gauze	Used to cover wounds
		Gloves	Used when changing wound dressings
		MediHoney®	Has antimicrobial properties and helps promote wound healing
		Triple antibiotic ointment	Helps to prevent or treat minor bacterial skin infections
All kits		Hand sanitizer	Reduces risk of infection

*Costs calculated by unit price of each kit content. Costs are an estimate and may vary based on vendors and supply availability

^†^Boofing refers to per-rectal administration of substances

^‡^Needles were not included in the safer injection kit but were prescribed separately using EHR preference lists to our partnering on-site institutional pharmacy. The default prescription sent were insulin needles 28G × 1/2", 1 cc, quantity 100, use as directed. Associated ICD-10 codes included Z20.6 (contact with and (suspected) exposure to HIV), Z20.2 (contact with and (suspected) exposure to infection with a predominantly sexual mode of transmission), or Z77.21 (contact with and (suspected) exposure to potentially hazardous body fluids) [27]. Substitutions allowed based on insurance coverage

^§^Due to Pennsylvania regulations related to drug paraphernalia [44], both safer smoking kits did not include stems or bowl pipes for crack cocaine or methamphetamine use, respectively. Patients were encouraged to visit the local SSP for provision of these supplies

#### Development of clinic workflow, workplace safety, and signage

During a pre-implementation staff meeting in December 2022, we identified a clinic staff champion, addressed potential barriers to implementation, and tailored a workflow with feedback from staff. We also designated a locked closet for centralized on-site kit storage and created a workplace safety plan in anticipation of patients bringing back used needles and other drug preparation equipment for disposal by installing sharps containers in all patient bathrooms as well as a discrete large-volume biohazard bin outside the clinic. Finally, to facilitate patient awareness of this QI program, we developed clinic posters communicating our clinic’s shared vision of “IM-REP = harm reduction” and created a patient-facing menu (Additional file 1: Fig. S2) featuring available kits that would be universally offered to patients presenting for in-person visits. Per our clinical workflow, this menu was distributed to every patient presenting for an in-person visit during check-in. Nurses reviewed the completed menu and provided requested kits to the patient in discrete bagging during the rooming process. Clinicians answered any additional questions about the clinic initiative, harm reduction in general, or kit-specific contents.

### Pilot program implementation

The pilot began in February 2023. Authors MS and RJ and the clinic champion provided weekly technical assistance for clinic staff if any supply procurement, clinical workflow, distribution, or occupational safety issues arose. Based on clinic staff consensus, patients could receive up to two of each kit per in-person clinic visit.

### Data sources

We conducted a one-month post-implementation program evaluation, measuring the number of patient-facing menus distributed and total kits requested and distributed to patients. De-identified data were collated using Microsoft Excel, stored on the institution’s secure file hosting service, and presented in aggregate. We evaluated implementation outcomes of program feasibility, acceptability, and appropriateness using an anonymous nine-question Likert scale staff/clinician survey based on the Implementation Outcomes Framework [28]. Staff/clinician surveys were distributed via email and administered via Qualtrics. Lastly, we assessed patient attitudes and satisfaction with our pilot program using an anonymous Qualtrics patient survey of multiple choice, open-ended, and Likert scale questions, the latter of which were derived and adapted from the Patient Assessment of Providers Harm Reduction Scale, which was initially developed for PWUD receiving HIV clinical care and is currently ongoing psychometric evaluation [29]. All Likert items were measured on a 5-point scale (1 = strongly disagree, 5 = strongly agree), and descriptive results were presented in aggregate. Patients were recruited on-site with fliers and were eligible for survey participation if they were 18 years of age or older and had received at least one type of kit since pilot implementation. If eligible and interested, patients completed the survey on-site using a clinic-provided laptop. Patients were compensated with a US$5 gift card for survey completion.

## Results

### Kit utilization

One month post-implementation, 28% (40/141) of patients who received a menu during an in-person visit requested at least one kit. Sixteen percent (23/141) of those who used the menu were classified as new patient visits, defined as establishing addiction care at our clinic, and of those, 30.4% (7/23) requested at least one kit. There were a total of 72 kit requests and 121 kits distributed as patients could receive up to two of each kit. The most frequently distributed kits included wound care (49/121), fentanyl test strips (23/121), and safer smoking (16/121).

### Implementation outcomes

Eight out of 10 staff and 10 out of 11 clinicians responded to our survey. All clinical respondents found the pilot program highly feasible, acceptable, and appropriate (see Additional file 1: Table S2). The majority of clinicians noted that their patients seemed happy to receive kits and having kits on-site provided them an opportunity to discuss safer use best practices and strategies for risk mitigation with patients. In contrast, one clinician stated that since many of their patients in their panel were in stable recovery, they did not require kits. The primary barrier to implementation was the inability to provide kits during telemedicine encounters. The majority of staff and clinicians responded that additional facilitating measures for future iterations of this pilot program included additional social support for patients (e.g., transportation), more stable sources of funding for kits, healthcare system policies supporting the intervention, and a formal partnership with the local SSP.

### Patient perceptions

Among the 30 patients who responded to our survey, most had positive perceptions of our pilot program even if they did not receive a kit. Most described that universally receiving a harm reduction menu at the time of checking into the clinic made them feel “*good*,” “*safe*,” and “*thankful*,” and none reported feeling uncomfortable or stigmatized. One patient stated, “*it made me feel that others were considering positive and alternative ways to help myself and others*,” and another patient said, “*it made me feel safe and accepted. I think too many times former heavy drug users are looked down on and their quality of life is not valued*.” The majority of patients also felt positively regarding their relationship with their addiction provider (see Additional file 1: Table S3).

## Discussion

We describe the successful implementation of a QI pilot program integrating kits in our OBAT clinic. Our program extends prior work in hospital settings domestically in the US [30] and internationally [23, 24] and serves as a real-world example addressing calls for harm reduction integration in outpatient care [7, 12, 26, 27]. Our findings, albeit limited to one-month post-implementation, highlight a significant unmet need for kits even among patients with access to long-term addiction treatment. These findings are consistent with our clinical experience treating patients with polysubstance use for which limited pharmacologic modalities are available outside of MOUD. Furthermore, even for those who take MOUD, patients may have periods of treatment discontinuation and/or intermittent use during which access to kits would be essential to mitigate risk of drug-related harms [31].

Given known barriers of healthcare-related stigma [32, 33] and lack of standardized medical education on harm reduction practices [34], we found that coupling clinician and staff training with on-site kit distribution facilitated the adoption of our intervention as a new clinic standard of care. We used multiple implementation strategies to communicate a shared vision to change our clinic culture to embrace a patient-centered approach to care grounded in the principles of relational harm reduction [35]. We also relied heavily on staff support and participation in developing and streamlining a clinic workflow and elicited frequent staff input and feedback during the pre- and post-implementation phases to promote an iterative process. As a result, clinicians and staff found our project to be highly feasible, appropriate, and acceptable. Finally, to address potential patient-anticipated stigma [33], we decided to universally provide our menu to all patients to normalize offering in-clinic harm reduction services irrespective of interest or stage of recovery and promote patient autonomy by bypassing the need to disclose any kit requests directly to clinicians. This was reinforced by our findings that patients felt our pilot program helped them feel accepted and safe and clinicians felt they had an opportunity to cultivate patient rapport and trust and bring up discussions about risk mitigation strategies even when patients did not disclose ongoing use.

Despite our successful pilot program, we encountered a number of implementation challenges. First, given our OBAT operates as a hybrid telehealth model, we were unable to systematically distribute kits to patients receiving care primarily via telemedicine during the pilot period. Future iterations of this program may explore and incorporate strategies to provide tele-harm reduction services, which have been described in some studies [36, 37]. Secondly, procurement of supplies and maintenance of kit inventory was difficult due to heterogeneity of vendors and pricing of kit supplies, unforeseen supply chain shortages, and unexpected variations in patient demands for specific kits. Through this experience, we learned about the importance of having a designated clinic champion closely monitor kit inventory as well as soliciting patient feedback to better understand fluctuations in kit requests. Lastly, finding sustainable sources for funding kit supplies was problematic and highlighted the need to explore diversified funding sources for supplies and mechanisms to allow for billing of the provision of these services.

## Conclusion

We found that integrating kits in the OBAT setting is a feasible and important step toward increasing patient access and utilization of life-saving harm reduction services and challenges the traditional model of addiction care. Our program uncovered a significant unmet need for people engaged in addiction treatment and reinforces the concept of a health behaviors continuum rather than a binary set of choices. Further research is needed to understand how such programs impact downstream patient clinical outcomes and cost-effectiveness, as well as how to best tailor implementation strategies to other OBAT settings including those with hybrid telemedicine models. Our description of the successful implementation of kits in our OBAT can serve as a framework for other outpatient addiction treatment programs in the US as we collectively seek to promote the health of PWUD.

### Supplementary Information

**Additional file 1.** Sample patient-facing educational pamphlet, menu, and program survey data.

## Data Availability

Not applicable.

## Abbreviations

PWUD: People who use drugs
SSP: Syringe service program
OBAT: Office-based addiction treatment
MOUD: Medications for opioid use disorder
QI: Quality improvement
IM-REP: Internal Medicine-Recovery Engagement Program
UPMC: University of Pittsburgh Medical Center
SUD: Substance use disorder

## References

[1] Spencer MR, Minino AM, Warner M (2022). Drug overdose deaths in the United States, 2001–2021. NCHS Data Brief.

[2] Ciccarone D (2019). The triple wave epidemic: supply and demand drivers of the US opioid overdose crisis. Int J Drug Policy.

[3] Gupta R, Holtgrave DR, Ashburn MA (2023). Xylazine—medical and public health imperatives. N Engl J Med.

[4] Ciccarone D, Unick GJ, Cohen JK, Mars SG, Rosenblum D (2016). Nationwide increase in hospitalizations for heroin-related soft tissue infections: associations with structural market conditions. Drug Alcohol Depend.

[5] Cicero TJ, Ellis MS, Kasper ZA (2020). Polysubstance use: a broader understanding of substance use during the opioid crisis. Am J Public Health.

[6] Schranz AJ, Fleischauer A, Chu VH, Wu LT, Rosen DL (2019). Trends in drug use-associated infective endocarditis and heart valve Surgery, 2007 to 2017: a study of statewide discharge data. Ann Intern Med.

[7] Actions Taken by the Biden-⁠Harris Administration to Address Addiction and the Overdose Epidemic 2022. https://www.whitehouse.gov/ondcp/briefing-room/2022/08/31/actions-taken-by-the-biden-harris-administration-to-address-addiction-and-the-overdose-epidemic/.

[8] Centers for Disease Control and Prevention. Summary of Information on the Safety and Effectiveness of Syringe Services Programs (SSPs) [updated January 11, 2023]. https://www.cdc.gov/ssp/syringe-services-programs-summary.html.

[9] Broz D, Carnes N, Chapin-Bardales J, Des Jarlais DC, Handanagic S, Jones CM (2021). Syringe services programs' role in ending the HIV epidemic in the U.S.: why we cannot do it without them. Am J Prev Med.

[10] Fernandez-Vina MH, Prood NE, Herpolsheimer A, Waimberg J, Burris S (2020). State laws governing syringe services programs and participant syringe possession, 2014–2019. Public Health Rep.

[11] Ijioma SC, Pontinha VM, Holdford DA, Carroll NV (2021). Cost-effectiveness of syringe service programs, medications for opioid use disorder, and combination programs in hepatitis C harm reduction among opioid injection drug users: a public payer perspective using a decision tree. J Manag Care Spec Pharm.

[12] Jawa R, Tin Y, Nall S, Calcaterra SL, Savinkina A, Marks LR (2023). Estimated clinical outcomes and cost-effectiveness associated with provision of addiction treatment in US primary care clinics. JAMA Netw Open.

[13] Weinstein ZM, Kim HW, Cheng DM, Quinn E, Hui D, Labelle CT (2017). Long-term retention in Office Based Opioid Treatment with buprenorphine. J Subst Abuse Treat.

[14] Krawczyk N, Allen ST, Schneider KE, Solomon K, Shah H, Morris M (2022). Intersecting substance use treatment and harm reduction services: exploring the characteristics and service needs of a community-based sample of people who use drugs. Harm Reduct J.

[15] Thakarar K, Nenninger K, Agmas W (2020). Harm reduction services to prevent and treat infectious diseases in people who use drugs. Infect Dis Clin N Am.

[16] Lee JD, Nunes EV, Novo P, Bachrach K, Bailey GL, Bhatt S (2018). Comparative effectiveness of extended-release naltrexone versus buprenorphine-naloxone for opioid relapse prevention (X:BOT): a multicentre, open-label, randomised controlled trial. Lancet.

[17] Krupitsky E, Nunes EV, Ling W, Illeperuma A, Gastfriend DR, Silverman BL (2011). Injectable extended-release naltrexone for opioid dependence: a double-blind, placebo-controlled, multicentre randomised trial. Lancet.

[18] Larochelle MR, Bernson D, Land T, Stopka TJ, Wang N, Xuan Z (2018). Medication for opioid use disorder after nonfatal opioid overdose and association with mortality: a cohort study. Ann Intern Med.

[19] Mattick RP, Breen C, Kimber J, Davoli M (2003). Methadone maintenance therapy versus no opioid replacement therapy for opioid dependence. Cochrane Database Syst Rev.

[20] Neale J, Nettleton S, Pickering L (2011). What is the role of harm reduction when drug users say they want abstinence?. Int J Drug Policy.

[21] Valente PK, Bazzi AR, Childs E, Salhaney P, Earlywine J, Olson J (2020). Patterns, contexts, and motivations for polysubstance use among people who inject drugs in non-urban settings in the US Northeast. Int J Drug Policy.

[22] National Health Care for the Homeless Council. Health Centers and Syringe Services Programs 2023. https://nhchc.org/wp-content/uploads/2023/06/Health-Centers-SSPs-Final.pdf.

[23] Miskovic M, Chan Carusone S, Guta A, O'Leary B, dePrinse K, Strike C (2018). Distribution of harm reduction kits in a specialty HIV hospital. Am J Public Health.

[24] Nolan S, Kelian S, Kerr T, Young S, Malmgren I, Ghafari C (2022). Harm reduction in the hospital: an overdose prevention site (OPS) at a Canadian hospital. Drug Alcohol Depend.

[25] Dogherty E, Patterson C, Gagnon M, Harrison S, Chase J, Boerstler J (2022). Implementation of a nurse-led overdose prevention site in a hospital setting: lessons learned from St. Paul's Hospital, Vancouver, Canada. Harm Reduct J.

[26] Taylor JL, Johnson S, Cruz R, Gray JR, Schiff D, Bagley SM (2021). Integrating harm reduction into outpatient opioid use disorder treatment settings: harm reduction in outpatient addiction treatment. J Gen Intern Med.

[27] Chatterjee A, Bannister M, Hill LG, Davis CS (2023). Prescribing syringes to people who inject drugs: advancing harm reduction in primary care. J Gen Intern Med.

[28] Proctor E, Silmere H, Raghavan R, Hovmand P, Aarons G, Bunger A (2011). Outcomes for implementation research: conceptual distinctions, measurement challenges, and research agenda. Adm Policy Ment Health.

[29] Kay ES, Creasy S, Batey DS, Coulter R, Egan JE, Fisk S (2022). Impact of harm reduction care in HIV clinical settings on stigma and health outcomes for people with HIV who use drugs: study protocol for a mixed-methods, multisite, observational study. BMJ Open.

[30] Perera R, Stephan L, Appa A, Giuliano R, Hoffman R, Lum P (2022). Meeting people where they are: implementing hospital-based substance use harm reduction. Harm Reduct J.

[31] Burns M, Tang L, Chang CH, Kim JY, Ahrens K, Allen L (2022). Duration of medication treatment for opioid-use disorder and risk of overdose among Medicaid enrollees in 11 states: a retrospective cohort study. Addiction.

[32] Tsai AC, Kiang MV, Barnett ML, Beletsky L, Keyes KM, McGinty EE (2019). Stigma as a fundamental hindrance to the United States opioid overdose crisis response. PLoS Med.

[33] Simon R, Snow R, Wakeman S (2020). Understanding why patients with substance use disorders leave the hospital against medical advice: a qualitative study. Subst Abus.

[34] Jawa R, Saravanan N, Burrowes SAB, Demers L (2021). A call for training graduate medical students on harm reduction for people who inject drugs. Subst Abus.

[35] Hawk M, Coulter RWS, Egan JE, Fisk S, Reuel Friedman M, Tula M (2017). Harm reduction principles for healthcare settings. Harm Reduct J.

[36] Davitadze A, Meylakhs P, Lakhov A, King EJ (2020). Harm reduction via online platforms for people who use drugs in Russia: a qualitative analysis of web outreach work. Harm Reduct J.

[37] Torres-Leguizamon M, Favaro J, Coello D, Reynaud EG, Nefau T, Duplessy C (2023). Remote harm reduction services are key solutions to reduce the impact of COVID-19-like crises on people who use drugs: evidence from two independent structures in France and in the USA. Harm Reduct J.

[38] Peckham AM, Young EH (2020). Opportunities to offer harm reduction to people who inject drugs during infectious disease encounters: narrative review. Open Forum Infect Dis.

[39] Imtiaz S, Strike C, Elton-Marshall T, Rehm J (2020). Safer smoking kits for methamphetamine consumption. Addiction.

[40] Ivsins A, Roth E, Nakamura N, Krajden M, Fischer B (2011). Uptake, benefits of and barriers to safer crack use kit (SCUK) distribution programmes in Victoria, Canada—a qualitative exploration. Int J Drug Policy.

[41] Fernandez N, Towers CV, Wolfe L, Hennessy MD, Weitz B, Porter S (2016). Sharing of snorting straws and hepatitis C virus infection in pregnant women. Obstet Gynecol.

[42] NEXT Distro. Rectal Administration. https://nextdistro.org/resources-collection/rectal-administration-booty-bumping-boofing-plugging.

[43] Chan CA, Canver B, McNeil R, Sue KL (2022). Harm Reduction in Health Care Settings. Med Clin North Am.

[44] Commonwealth of Pennsylvania. The Controlled Substance, Drug, Device and Cosmetic Act [ ]. https://www.legis.state.pa.us/WU01/LI/LI/US/HTM/1972/0/0064..HTM.

