# Supplementary material for: Implementing harm reduction kits in an office-based addiction treatment program
Source: Harm Reduct J. 2023 Nov 2;20:163. doi: 10.1186/s12954-023-00897-5 (PMC10621216; doi:10.1186/s12954-023-00897-5)
Supplement: Supplementary file 1 — Additional file 1. Sample patient-facing educational pamphlet, menu, and program survey data. [file 12954_2023_897_MOESM1_ESM.docx]

**Appendix Figure 1.** Safer injection handout*

**
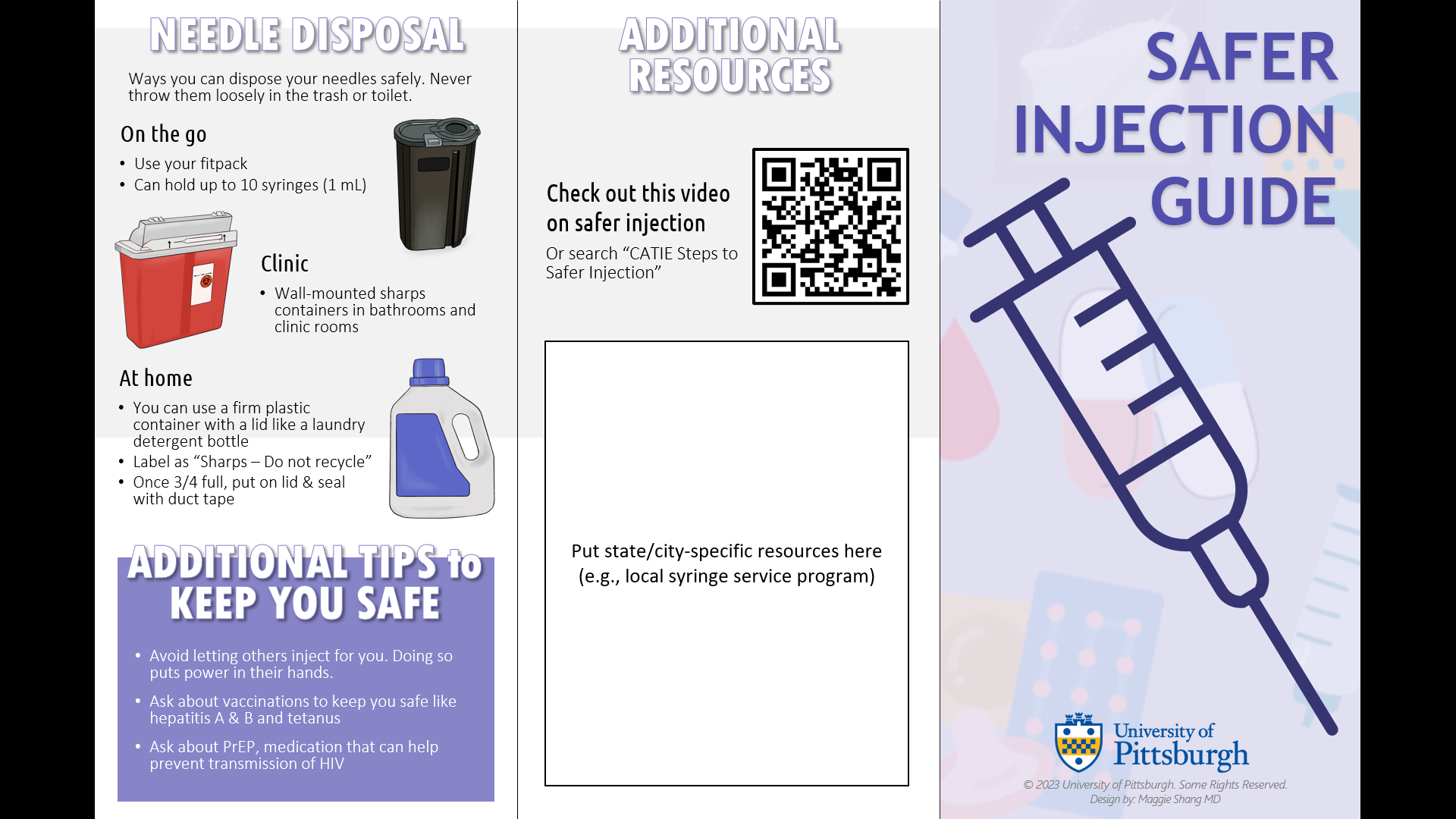
**

**
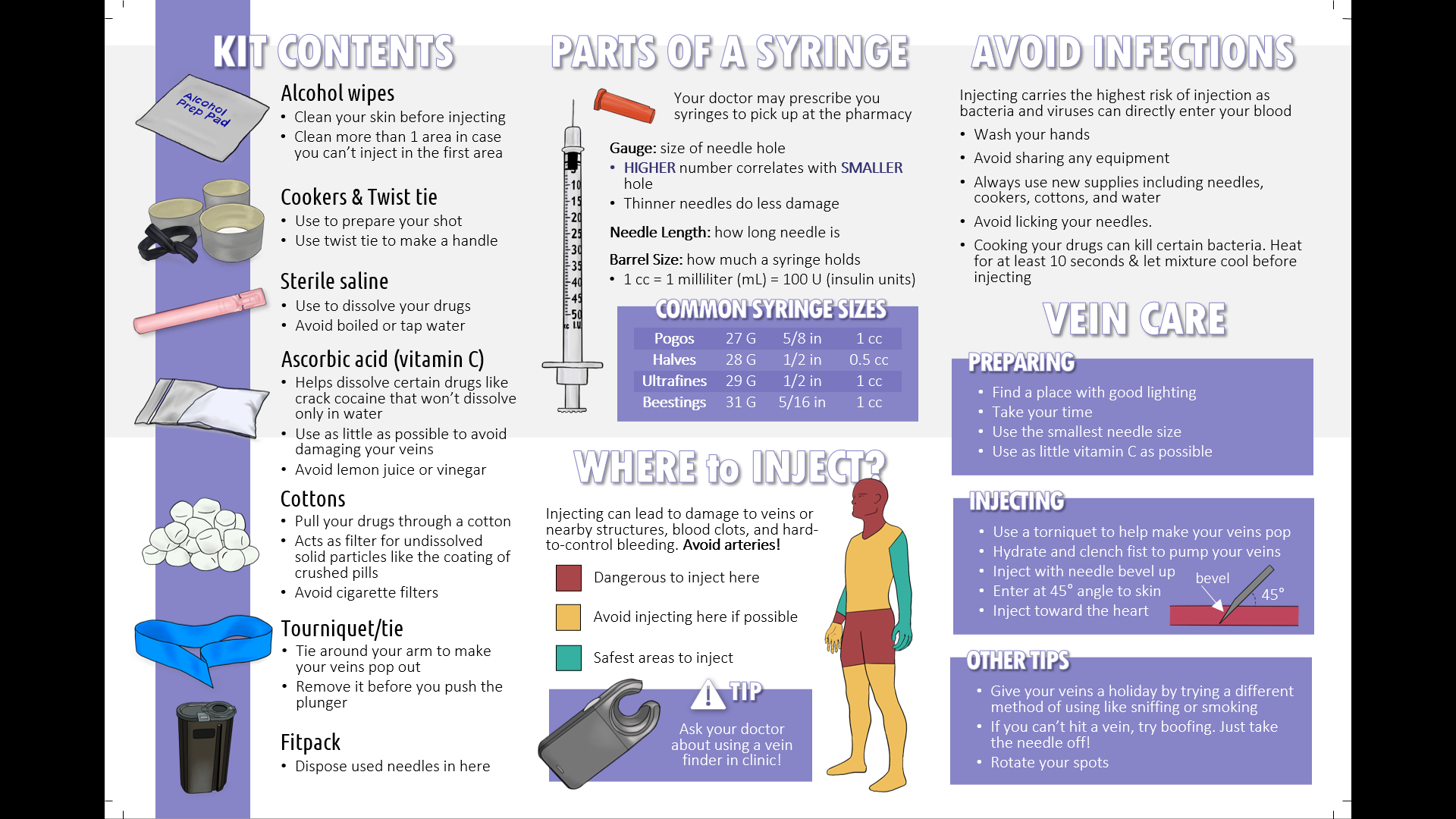
**

*Sample guide provided for manuscript. All pamphlets are made available via the Creative Commons License for adaptation and redistribution in the D-Scholarship Institutional Repository at the University of Pittsburgh including the following:

- Fentanyl Test Strip Guide: DOI: 10.18117/52mg-0132
- Safer Boofing Guide: DOI: 10.18117/7qjv-6h62
- Safer Injection Guide DOI: 10.18117/tgrb-ts13
- Safer Smoking Guide for Crack Cocaine: DOI: 10.18117/y20k-5d40
- Safer Smoking Guide for Methamphetamines: DOI: 10.18117/mhhd-za83
- Safer Snorting Guide: DOI: 10.18117/vemw-1f97
- Wound Care Guide: DOI: 10.18117/h786-g714

**Appendix Figure 2.** Patient-facing menu used to aid kit distribution


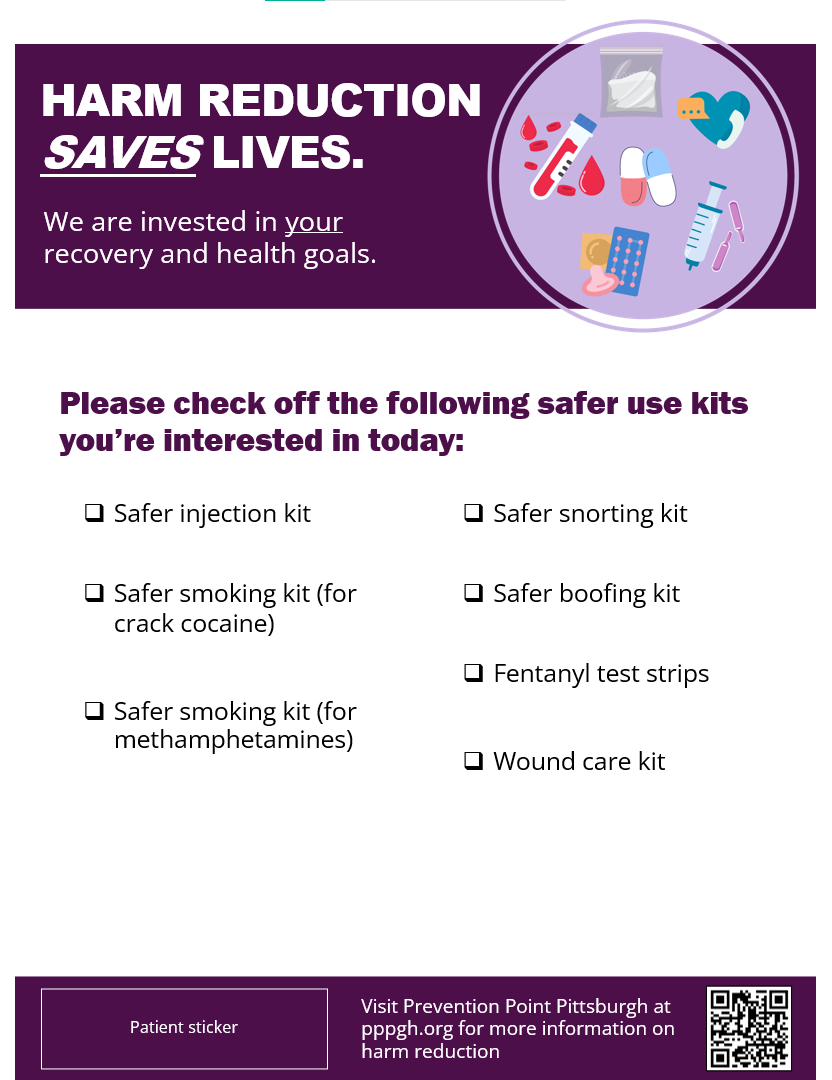


**Appendix Table 1.** Pre- and post-harm reduction training comfort items using a 5-point Likert scale (N = 10 staff)

| **Statement** | **Pre^a^ (mean)** | **Post^a^ (mean)** |
| --- | --- | --- |
| I feel comfortable administering naloxone to reverse an opioid overdose. | 3.0 | 3.5 |
| I feel comfortable distributing harm reduction kits within the clinic setting. | 3.4 | 4.0 |
| I have the knowledge to select the best type of harm reduction kit based on what my patient needs.^b^ | 3.0 | 4.6 |

^a^Outcomes were measured on a 5-point Likert scale where 1 is very uncomfortable and 5 is very comfortable.

^b^for p-value < 0.05.

**Appendix Table 2.** One-month post-program implementation outcomes of acceptability, appropriateness, and feasibility using a 5-point Likert scale (N = 18 clinicians and staff)

| **Implementation Outcome** | **Statement** | **Average^a^** |
| --- | --- | --- |
| Feasibility | I have enough time to participate. | 4.1 |
|  | We have enough staff to do this. | 4.6 |
|  | This fits into our workflow. | 4.6 |
|  | Communication within our clinic regarding kits works well. | 4.5 |
| Acceptability | I feel comfortable handing out kits. | 4.6 |
|  | Kits are a priority for my patients. | 4.6 |
|  | I perceive that patients irrespective of race feel comfortable requesting kits. | 4.3 |
| Appropriateness | This is part of my job. | 4.5 |
|  | I have appropriate skills to distribute and discuss kits with patients. | 4.2 |

^a^Outcomes were measured on a 5-point Likert scale where 1 is strongly disagree and 5 is strongly agree.

**Appendix Table 3.** Patient perceptions on relationship with their addiction provider one-month post-implementation using a 5-point Likert scale (N = 30 patient participants)

| **Statement** | **Average^a^** |
| --- | --- |
| I have an equal voice with my addiction provider in making decisions about my care. | 4.8 |
| My addiction provider helps me identify health goals that work for me. | 4.8 |
| My addiction provider respects me even if I have harmful health behaviors. | 4.7 |
| My addiction provider celebrates when I make positive health changes even if they are small changes. | 4.8 |
| My addiction provider understands that sometimes I make decisions based on quality of life rather than strict health outcomes. | 4.7 |
| My addiction provider helps me understand how my harmful behaviors might impact my health. | 4.9 |

^a^Outcomes were measured on a 5-point Likert scale where 1 is strongly disagree and 5 is strongly agree.
